# Individual and contextual factors associated with appropriate healthcare seeking behavior among febrile children in Tanzania

**DOI:** 10.1371/journal.pone.0175446

**Published:** 2017-04-13

**Authors:** Juma Adinan, Damian J. Damian, Neema R. Mosha, Innocent B. Mboya, Redempta Mamseri, Sia E. Msuya

**Affiliations:** 1 Assistant Medical Officer (AMO)-General Teaching College, KCMC Hospital, Moshi, Tanzania; 2 Institute of Public Health, Department of Community Health, Kilimanjaro Christian Medical University College (KCMU Co), Moshi, Tanzania; 3 Department of Community Health, Kilimanjaro Christian Medical Centre (KCMC) Hospital, Moshi, Tanzania; 4 Institute of Public Health, Department of Epidemiology and Biostatistics, Kilimanjaro Christian Medical University College (KCMU Co), Moshi, Tanzania; University of North Carolina at Chapel Hill School of Dentistry, UNITED STATES

## Abstract

**Introduction:**

Fever in malaria endemic areas, has been shown to strongly predict malaria infection and is a key symptom influencing malaria treatment. WHO recommended confirmation testing for *Plasmodium spp*. before initiation of antimalarials due to increased evidence of the decrease of morbidity and mortality from malaria, decreased malaria associated fever, and increased evidence of high prevalence of non-malaria fever. To immediately diagnose and promptly offer appropriate management, caretakers of children with fever should seek care where these services can be offered; in health facilities.

**Objectives:**

This study was conducted to describe healthcare seeking behaviors among caretakers of febrile under five years, in Tanzania. And to determine children’s, household and community-level factors associated with parents’ healthcare seeking behavior in health facilities.

**Methods:**

Secondary data analysis was done using the Tanzania HIV and Malaria Indicator Surveys (THMIS) 2011–2012. Three-level mixed effects logistic regression was used to assess children’s, household and community-level factors associated with appropriate healthcare seeking behavior among care takers of febrile children as well as differentiating between household and community variabilities.

**Results:**

Of the 8573 children under the age of five years surveyed, 1,675(19.5%) had a history of fever two weeks preceding the survey. Of these, 951 (56.8%) sought appropriate healthcare. Febrile children aged less than a year have 2.7 times higher odds of being taken to the health facilities compared to children with two or more years of age. (OR: 2.7; 95%CI: 1.50–4.88). Febrile children from households headed by female caretakers have almost three times higher odds of being taken to the health facilities (OR: 2.85; 95%CI; 1.41–5.74) compared to households headed by men. Febrile children with caretakers exposed to mass media (radio, television and newspaper) have more than two times higher odds of being taken to health facilities compared to those not exposed to mass media. Febrile children from regions with malaria prevalence above national level have 41% less odds of being taken to health facilities (OR: 0.49; 95%CI: 0.29–0.84) compared to those febrile children coming from areas with malaria prevalence below the national level. Furthermore, febrile children coming from areas with higher community education levels have 57% (OR: 1.57; 95%CI: 1.14–2.15) higher odds of being taken to health facilities compared to their counterparts coming from areas with low levels of community education.

**Conclusion and recommendation:**

To effectively and appropriately manage and control febrile illnesses, the low proportion of febrile children taken to health facilities by their caretakers should be addressed through frequent advocacy of the importance of appropriate healthcare seeking behavior, using mass media particularly in areas with high malaria prevalence. Multifaceted approach needs to be used in malaria control and eradication as multiple factors are associated with appropriate healthcare seeking behavior.

## Introduction

Fever in malaria endemic areas, has been shown to strongly predict malaria infection[1,2] and remains to be a key symptom influencing malaria treatment. Following the Roll-Back Malaria initiative in Tanzania, morbidity and mortality from malaria have declined significantly from the early eighties to 2014 [3–7]. Due to increased evidence of the decrease of morbidity and mortality from malaria, decreased malaria associated fever[2], and increased evidence of high prevalence of non-malaria fever [8,9], WHO recommended confirmation testing for *plasmodium spp*. before initiation of antimalarials [10].

It is now evident that, febrile children under five years of age need to be investigated for other non-malaria causes of fever. It has been shown that the proportion of fever as a predictor of malaria varies from place to place depending on malaria prevalence and there are significant proportions of patients with fever caused by other infections [7,8]. To timely diagnose and promptly offer appropriate management, caretakers of children with fever should seek care where these services are offered; at health facilities. Seeking appropriate care is advantageous to patients as it allows correct diagnosis to be made[8] and hence appropriate subsequent care[11] in addition to the collection of data for malaria surveillance and understanding the epidemiology of causes of fever other than malaria.

Despite the fact that the vast majority (90%) of Tanzanians live within 5 km of a health facility, only 40–54% of caretakers with febrile children seek healthcare [12–14]. This leaves a large proportion of children at risk of severe complications and death. Different factors influencing caretakers’ effort to seek health care of febrile children have been reported in different settings. These factors can be categorized as: caretakers social economic status, age and marital status, child’s; age and sex, effectiveness and adverse outcomes of previously administered medicines, a specific health facility; inadequate supply of medications and poor attitudes of healthcare providers and disease severity [15–25].

Studies on determinants of healthcare seeking behavior among caretakers with febrile children conducted in Tanzania focused on seeking care anywhere [13]. Other studies were done during a period when MRDT were not widely used and advocacy of appropriate healthcare seeking behavior was not scaled-up [12,13]. Subsequent to these activities there is limited information on the factors associated with appropriate healthcare seeking behaviors concerning children under five years of age.

This study was conducted to describe healthcare seeking behaviors among caretakers of febrile children under the age of five years in Tanzania, and to determine children’s, household and community-level factors associated with healthcare seeking behavior in health facilities. This is the first study with the national representative sample to describe appropriate healthcare seeking behavior as well as the influence of contextual factors on seeking appropriate healthcare for febrile children under five years of age.

## Methodology

Secondary analysis of data from Tanzania HIV and Malaria indicator survey (THMIS) 2011–2012 was done. The survey is the third and most recent in Tanzania. This is a national sample survey that aims to estimate the key indicators of malaria and HIV for each Tanzanian region. THMIS is a nationally-representative cross-sectional household survey that is performed every four years.

THMIS data can be generalized from the regional, zonal and national levels; not below these levels. Samples were obtained by using two stage sampling, selection of communities (clusters) and households. Communities were selected from a list of enumerated areas of the 2002 Population and Housing Census.

In individual questionnaires, children’s caretakers were interviewed to collect information on: socio-demographic characteristics, knowledge of malaria, the history of fever two weeks preceding the survey and, care seeking for treatment of fever. The Household Questionnaire was used to collect information on households to determine the wealth index[14].

In the 2011–12 THMIS, 583 communities were selected from 10,496 households that participated in the survey. In total, 8,648 caretakers with children under the age of five years were interviewed. The Children Records (KR dataset) was used for the analysis. The unit of analysis in this study was children under five (5) years old (age 6–59 months) and their caretakers.

### Variables

Due to the nature of the study, that children were nested within the household and that household are nested within the community, three sets of independent variables were created. The three sets are children’s, household and community-level factors. Children’s variables were: demographic characteristics of children (age and sex). Household variables were socio-demographic characteristics of caretakers (age, sex, marital status, occupation, educational level, wealth index), possession of health insurance, caretaker media exposure, caretaker perception of the capability of protecting his/her family from malaria and sex of the household leader.

Community-level variables included in the study are: residence; urban and rural, community education, community exposure to mass media, community wealth index and community malaria prevalence.

The dependent variable of the study was appropriate healthcare seeking behavior for febrile children under the age of five years in Tanzania. Appropriate healthcare seeking behavior was defined as taking a febrile child to health facilities for medical care within 48 hours of fever onset.

### Variable recoding

Community education variables were categorized into two categories: Communities with low and high education levels. The level of education was put into an ordinal scale: The level above overall national communities median scale was labeled community with high level of education and the levels within and below overall national communities median were labeled communities with low levels of education.

The community wealth index variable was put into two categories, communities with low and high socio-economic status. The wealth index was put into an ordinal scale. The level above median overall national communities scale was labeled communities with high socio-economic status and those within and below overall national communities median were labeled community with low socio-economic status.

Community malaria prevalence was compared with that of the national prevalence, nine (9%) [14]. Communities with malaria prevalence below that of national level were labeled communities with low malaria prevalence and those with malaria prevalence above the national level were labeled communities with high malaria prevalence.

Community exposure to mass media was put into two categories: communities with low and high mass media exposure. Mass media exposure was put into an ordinal scale, communities above overall national communities median scale were labeled communities with high mass media exposure and those within and below overall national communities median were labeled communities with low mass media exposure.

### Statistical analysis

Stata version 13 SE was used for analysis. Descriptions of children’s, household and community-level characteristics were presented in percentages. Sampling weight was used to account for non-response and disproportionate sampling.

As cluster-based data, Intra-class Correlation Coefficient (ICC) was checked before choosing the appropriate method of determining factors associated with appropriate healthcare seeking behavior among caretakers with children under five years of age. The ICC was checked because there might be differences in appropriate healthcare seeking behaviors between communities surveyed as well as similarities within a cluster. The ICC was 27% which necessitated multilevel analysis. To address this, three-level mixed effects logistic regression was used to assess children’s, household and community-level factors associated with appropriate healthcare seeking behavior among caretakers with children with fever as well as determining between cluster (community) variability of levels of appropriate healthcare seeking behavior. The first level of analysis was the individual level and the second was the cluster level. Likelihood ratio testing was used to test the goodness of fit and to select the final parsimonious model.

### Ethical clearance

Before data collection, the overall aim of the survey was explicitly communicated to participants and signed written informed consent was sought[14]. Participants’ identification numbers were used to preserve confidentiality. Permission to use this data was obtained from the DHS PROGRAM. The ethical clearance to conduct this study was obtained from Kilimanjaro Christian Medical University College (KCMU Co), Kilimanjaro, Tanzania. Participants’ records were anonymized and de-identified by the DHS-Program before dataset was released to the public domain.

## Results

### Baseline characteristics of participants

Thirty (30) strata were involved in this study, from these, 583 communities were surveyed and caretakers of 8,573 (weighted number) children were interviewed. Of the 8,573 children, the majority were female 4313 (50.3%). The mean age of children was 30.9±15.6 months, the majority with or more than 24 months of age.

The majority of caretakers were age 15–49 (63.7%), had attained primary education (66.4%) and were married (84.5%). In addition, majority of the caretakers were not working (88.4%), not covered with health insurance (92.9%) and perceived being capable of protecting their families from malaria infection (93.8%). The majority of households were headed by males (82.9%), had low socio-economic status (45.6%), and with caretakers exposed to at least one form of mass media.

The majority (61.8%) of children came from regions with lower malaria prevalence compared to that of the nation, and from rural areas (82.6%). There were slight differences in percentage distributions of children by community wealth index, community media exposure and community educational level Table 1.

**Table 1:** Baseline characteristics and prevalence of fever among children by children’s, household and community-level factors.

Participants characteristics	Distribution of participants’ characteristics (n = 8573)	Children who had fever two weeks preceding the (n = 1675)
	Number. (%)	Number (%)
**Child sex**		
*Male*	4260 (49.7)	854 (20.9)
*Female*	4313 (50.3)	821 (19.9)
**Children’s Age in months** ^**m**^		
*06–11*	881 (12.7)	256 (29.1)
*12–23*	1759 (25.3)	509 (28.9)
*> = 24*	4318 (62.1)	774 (17.9)
**Education**		
*No*	2152 (25.1)	372 (18)
*Primary*	5691 (66.4)	1158 (21.2)
*Secondary*	230 (8.5)	145 (20.9)
**Marital status**		
*Single*	489 (5.7)	97 (20.7)
*Married*	7243 (84.5)	1393 (20)
*Separated*	841 (9.8)	184 (23.5)
**Working** ^**m**^		
*No*	7580 (88.5)	1501 (20.7)
*Yes*	988 (11.5)	173 (18.3)
**Covered by health insurance** ^**m**^		
No	7965 (92.9)	1568 (20.6)
Yes	583 (6.8)	100 (17.4)
**Wealth**		
Poorest	1963 (22.9)	399 (21.3)
Poorer	1947 (22.7)	391 (20.8)
Middle	1697 (19.8)	304 (18.5)
Richer	1566 (18.3)	289 (19.3)
Richest	1401 (16.3)	292 (22)
**Caretakers’ age categories**		
*15–44*	3114(36.3)	616 (20.8)
*45–64*	3683 (43)	725 (20.5)
*65+*	1776 (20.7)	334 (19.4)
**Exposure to media (Television, Radio, Newspaper)**		
No exposure	1764 (20.6)	319 (18.8)
Exposed to one	3149 (36.7)	650 (21.4)
Exposed to two	2310 (26.9)	458 (20.8)
Exposed to three	1350 (15.7)	249 (19.4)
**Caretakers’ perception on capability of protection of self and family from malaria**		
Cannot protect	533 (6.2)	125 (24.6)
Can protect	8040 (93.8)	155 (20.1)
**Sex of head of household**		
Male	7105 (82.9)	1396 (20.5)
Female	1468 (17.1)	-lev279 (19.9)
**Malaria prevalence in comparison with national prevalence**		
Low	5297 (61.8)	820 (16.1)
High	3276 (38.2)	855 (27.3)
**Residence**^**m**^		
*Urban*	1496 (17.5)	316 (22.3)
*Rural*	7076 (82.5)	1359 (20)
**Community education level**		
Low Education	4152 (48.4)	820.5(20.5)
High Education	4421 (51.6)	854.8(20.3)
**Community wealth index**		
Low	4531 (52.8)	865 (19.8)
High	4042 (47.2)	810 (21)
**Community media exposure**		
low	4079 (47.6)	792 (20.2)
High	4494 (52.4)	883 (20.6)

^m^ Missing values

### Prevalence of fever among under-fives by social demographic characteristics

Of the 8, 573 children under the age of five years surveyed, 1675(19.5%) had a history of fever two weeks preceding the survey. Of the 1,675 children under the age of five years with fever, 29.1% were children younger than one year old. Almost equal proportion of children classified by sex had fever i.e. 20.9% and 19.9% respectively. Table 1 shows that the prevalence of fever varies slightly between other different individual and community-level factors of participants.

### Healthcare seeking behavior

**Fig 1:**
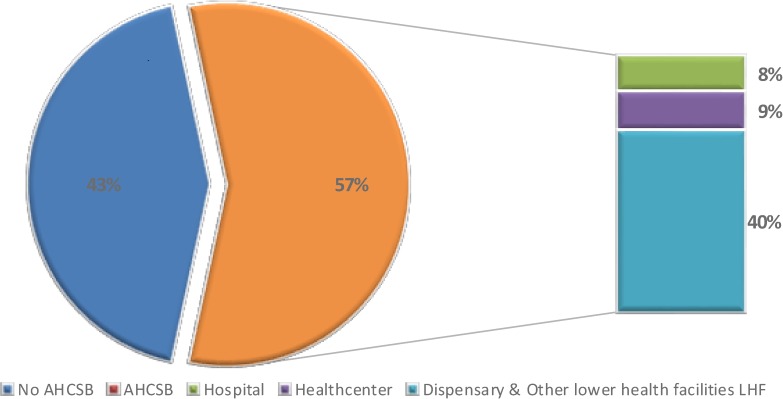
Description of healthcare facilities sought by caretakers of children under five years of age with fever, two weeks preceding the survey.

Of the 8,216 who responded to the question on the fever status of the child two weeks preceding the survey, 1,675 (20.4%) had a history of fever two weeks preceding the survey. Of the 1,675, 951 (56.8%) were taken to healthcare facilities (sought appropriate care). Of the 951 who sought appropriate healthcare, 131 (7.8%), 144 (8.6%) and 676 (40.4%) sought care at hospitals, health centers and dispensaries respectively as shown in Fig 1. Of the 724 that did not seek appropriate healthcare; 391 (23.3%) bought medication from drug outlets and 333 (19.9%) did not seek healthcare at all. Of the 951 who sought appropriate healthcare, 394 (41.5%) were tested for malaria. Of those tested, 291 (78.9%) were diagnosed with malaria. Table 2 describes healthcare seeking behavior among caretakers with children with fever two weeks preceding the survey.

**Table 2:** Appropriate healthcare seeking behavior among caretakers with children under five years of age who had fever two weeks preceding the survey; by children’s, household and community-level factors (n = 1675).

Characteristics	Febrile Children	Appropriate Healthcare Seeking Behavior	
		No	Yes
	Number (1675)	Number (%)	Number (%)
**Child sex**			
*Male*	854	345 (40.4)	509 (59.6)
*Female*	821	379 (46.2)	442 (53.8)
**Children’s Age in months**^**m**^			
*06–11*	256	86 (33.5)	170 (66.5)
*12–23*	509	225 (44.2)	284 (55.8)
*> = 24*	774	361 (46.6)	413 (53.4)
**Caretakers’ Education**			
*No*	372	199 (53.5)	173 (46.5)
*Primary*	1158	496 (42.8)	662 (57.2)
*Secondary*	146	30 (20.4)	116 (79.6)
**Marital status**			
*Single*	97	24 (25.1)	73 (74.9)
*Married*	1395	617 (44.3)	777 (55.7)
*Separated*	184	83 (45.1)	101 (54.9)
**Working**^**m**^			
*No*	1501	675 (45)	826 (55)
*Yes*	173	48 (28)	125 (72)
**Covered by health insurance**			
No	1568	694 (44.3)	874 (55.7)
Yes	100	29 (28.7)	72 (71.3)
**Wealth index**			
Poorest	399	199 (49.8)	201 (50.2)
Poorer	391	207 (52.9)	184 (47.1)
Middle	304	141 (46.3)	163 (53.7)
Richer	289	108 (37.4)	181 (62.6)
Richest	292	70 (24)	222 (76)
**Caretakers’ age categories**			
*15–44*	616	252 (40.9)	364 (59.1)
*45–64*	725	312 (43)	413 (57)
*65+*	335	161 (48.1)	174 (51.9)
**Exposure to media (Television, Radio, Newspaper)**			
No exposure	319	192 (60.1)	127 (39.9)
Exposed to one	650	297 (45.6)	354 (54.4)
Exposed to two	457	176 (38.6)	280 (61.4)
Exposed to three	249	60 (24)	190 (76)
**Caretaker can protect self and family from malaria**			
Cannot protect	125	73 (58.8)	51 (41.2)
Can protect	1551	651 (42)	900 (58)
**Sex of head of household**			
Male	1397	623 (44.6)	773 (55.4)
Female	279	101 (36.3)	178 (63.7)
**Malaria prevalence in comparison with national prevalence**			
Low	820	324 (39.5)	496 (60.5)
High	856	401 (46.9)	455 (53.1)
**Residence**			
Urban	316	81 (25.7)	235 (74.3)
Rural	1359	643 (47.3)	716 (52.7)
**Community education level**			
Low Education	821	452 (55)	369 (45)
High Education	855	273 (31.9)	582 (68.1)
**Community wealth index**			
Low	865	449 (51.9)	416 (48.1)
High	811	276 (34.1)	535 (65.9)
**Community media exposure**			
low	792	412.8(52.1)	380 (47.9)
High	883	311.6(35.3)	571 (64.7)

^m^ Missing values

### Multivariable multilevel analyses

A three-level mixed effects logistic regression model was used to analyse the effects of child, house-hold and community-level factors in care givers’ of febrile children decision on healthcare seeking behaviour. In an empty model, a model without observed characteristics, 36.14% of the total variance in the odds seeking appropriate healthcare was accounted for by between-community variation of characteristics (ICC = 0.27, p<0.0001) while 43.70% was due to between–household variation of characteristics (ICC = 0.4370, p<0.0001). Both between-community and household variability declined over successive models. The declines were from 36.14% in the empty model to 29.28% in model combining child, household and community factors for the community variability and from 43.70% in the empty model to 43.57% in model combining individual, household and community factors for the household variability (Table 3). The combined model of child-level, household and community-level factors was selected for predicting caretakers’ decision about healthcare seeking behaviour.

**Table 3:** Adjusted analysis of factors associated with appropriate healthcare seeking behavior among caretakers with children under five years of age, with fever.

*Variables*	Individual factors Model	Household factors Model	Community factors Model	Individual, Household, Community factors Model
Sex of child	** **	** **	** **	** **
*Male*	1	** **	** **	1
*Female*	0.75[0.48–1.17]			0.73[0.48–1.10]
*Child age category*				
*<12*	2.88***[1.55–5.34]			2.70***[1.50–4.88]
*23-Dec*	1.13[0.70–1.82]			1.13[0.72–1.78]
*24+*	1			1
***Household factors***					
Caretakers' education				
*No*		1		1
*Primary*		1.13[0.66–1.93]		1.10[0.63–1.91]
*Secondary+*		2.36[0.94–5.90]		1.47[0.56–3.84]
Caretakers' marital status				
*Never married*		1		1
*Married*		1.02[0.38–2.74]		0.82[0.28–2.38]
*Separated*		0.53[0.17–1.61]		0.44[0.13–1.46]
Working				
*Yes*		1		1
*No*		2.07[1.00–4.31]		1.74[0.79–3.84]
Covered by health insurance				
*No*		1		1
*Yes*		1.78[0.70–4.49]		1.84[0.72–4.66]
Wealth index				
*Poorest*		1		1
*Poorer*		0.76[0.40–1.43]		0.78[0.41–1.49]
*Middle*		0.87[0.45–1.70]		0.67[0.33–1.33]
*Richer*		1.44[0.71–2.90]		1.01[0.47–2.14]
*Richest*		2.32[0.94–5.75]		1.33[0.44–4.04]
Caretakers' age categories				
*15–25*		1		1
*26–35*		1.96*[1.09–3.53]		1.67[0.91–3.05]
*36–49*		1.74[0.98–3.08]		1.43[0.81–2.53]
Caretaker can protect self and family from malaria				
*No*		1		1
*Yes*		1.77[0.80–3.94]		2.19[0.95–5.07]
Sex of head of household				
*Male*		1		1
*Female*		2.91**[1.46–5.78]		2.85**[1.41–5.74]
Exposure to TV, radio, and Newspaper				
*No exposure*		1		1
*Exposed to one*		2.46**[1.34–4.53]		2.95***[1.56–5.61]
*Exposed to two*		2.46*[1.23–4.94]		2.77**[1.33–5.78]
*Exposed to three*		4.48***[1.85–10.82]		5.09***[2.02–12.86]
**Community-level factors**				
Residence				
*Rural*			1	1
*Urban*			0.55[0.26–1.17]	1.10[0.44–2.73]
Malaria prevalence in comparison with national prevalence				
*High*			1	1
*Low*			0.46**[0.27–0.77]	0.49**[0.29–0.84]
*Community education level*				
*Low Education*			1	1
*High Education*			3.02***[1.65–5.54]	2.29**[1.25–4.19]
Community poverty level				
*Low*			1	1
*High*			0.54*[0.30–0.96]	0.56[0.30–1.04]
Community media exposure				
*low*			1	1
*High*			1.06[0.59–1.89]	0.86[0.48–1.56]
* *				
ICC Community	36.18	31.5	29.11	27.14
ICC Household	44.73	42.16	42.76	42.15

Significant at *** p<0.001; ** p<0.01

^1^ Reference category

### Children’s factors associated with appropriate healthcare seeking behavior

In adjusted analysis as shown in Table 3: children factors found to be independently and statistically significantly associated with appropriate healthcare seeking behaviors is child’s age. Febrile children aged less than a year have 2.7 times higher odds of being taken to the health facilities compared to children two or more years of age. (OR: 2.7; 95%CI: 1.50–4.88).

### Household factors associated with appropriate healthcare seeking behavior

Caretakers’ sex and caretakers’ exposure to mass media were found to be statistically and significantly associated with appropriate healthcare seeking behaviors. Febrile children from households headed by female caretakers have almost three times higher odds of being taken to the health facilities (OR: 2.85; 95%CI; 1.41–574) compared to households headed by men. In addition, children with caretakers exposed to mass media have more than two times higher odds of being taken to appropriate health care facilities. Mass media studied were newspapers, radio, and television. Children with caretakers exposed to one of these mass media, exposed to two of these and all of these have two times higher odds (OR: 2.95; 95%CI: 1.56–5.61), have more than two times higher odds 2.77 (1.31–5.78) and more than 5 times higher odds of being taken to health facilities (OR: 3.11: 95%CI: 2.02–12.86) respectively compared to those not exposed to media.

### Community factors associated with appropriate healthcare seeking behavior

Community factors found to be independently and statistically significantly associated with appropriate healthcare seeking behavior are community malaria prevalence and community education level. Febrile children from regions with malaria prevalence above the national level have less odds of being taken to appropriate health care facilities (OR: 0.64; 95%CI: 0.48–0.86) compared to those febrile children coming from areas with malaria prevalence below the national level. Furthermore, febrile children coming from areas with higher community education levels have more than two (OR: 2.29; 95%CI: 1.25–4.19) times higher odds of being taken to health facilities compared to their counterparts coming from areas with low education levels as shown in Table 3.

## Discussion

This study determined child, household and community-level factors associated with appropriate healthcare seeking behavior. Results show that there are still a substantial number of febrile children under the age of five years that are not taken to health care facilities. Furthermore, results showed child’s factors associated with appropriate healthcare seeking behaviors among caretakers to be child’s age. Sex of the household head and caretaker’s exposure to mass media were household factors that are associated with appropriate healthcare seeking. Also, this study showed community-level factors associated with appropriate healthcare seeking behavior among caretakers to be community malaria prevalence and community education level.

The study’s result shows that only 56.8% of febrile children are taken to appropriate healthcare facilities by their caretakers, 391 (23.3%) bought medication from drug outlets and 333 (19.9%) did not seek healthcare at all. This means 43.2% of febrile children may not only suffer from complicated malaria if fever is *P*.*falciparum* malaria associated but also contributing to malaria transmission [1]. Perhaps caretakers are taking children with fever for care outside the health care facilities due to the perception that medications are not available at facilities[15] and so decide to seek care somewhere else, for example at the pharmacies. This underscores the importance of further advocacy and community mobilization on appropriate healthcare seeking for febrile children. These results are similar to that reported in Kilosa, Tanzania, and in Dhaka, Bangladesh[12,17]. However, there is a substantial discrepancy in the proportion of febrile children taken to appropriate healthcare facilities reported by other researchers from Southern Ghana (11%), Ethiopia (14%), Kenya (29.3%), Southern Sudan (39%), Senegal (40%) and Tanzania (77%) [14,23–29]. These differences might be accounted for by the study sites, study population involved and the analysis. The study conducted in Kenya involved the poorest population while those of Sudan, Ghana and Ethiopia involved only rural dwellers and in the study conducted in Tanzania, analysis included caretakers who went to pharmacies and informal sectors to seek care.

Caretakers exposed to more than one type of mass media have higher odds of seeking appropriate care when they have febrile children. Messages on malaria prevention and management are being delivered through mass media. Key messages being delivered are: consistent use of insecticide treated nets (ITN), importance of malaria testing when having fever before using antimalarials, emphasizing the fact that not every fever is malaria related “*si kila homa ni malaria*” and effectiveness of and adherence to, Artemisinin Combined Therapy (ACT) in malaria treatment. Access to mass media exposes caretakers to these key messages on malaria management, seeking care at the health facility being among those. This finding shows the important role mass media play in health promotion. Mass media have been shown to have similar positive impact in health facility deliveries [28] and contraceptive use.

Education has been associated with appropriate healthcare seeking behavior in different countries for various health services. This study showed that children coming from communities with levels of education above national averages have higher odds of being taken to appropriate health care facilities, compared to those from the communities with lower education. This finding is in line with that reported in Ethiopia and Tanzania [13,29]. Literacy and knowledge of malaria have been shown to be associated with the attitude that malaria is treatable [30]. Education is not only associated with care seeking for children under five years of age with fever, but has also shown to influence use of health facilities and skilled attendants during pregnancy, childbirth, the postnatal period for women and children as well as having higher vaccination coverage and better nutritional status for children[31]. Being within communities with an educated majority exposes the caretakers with febrile children to people who are capable of analyzing key health information [30] and so advocate for appropriate care seeking.

Low odds of healthcare seeking behavior among care takers of febrile children in areas with prevalence of malaria above the national level could be explained by the fact that there are more instances of self-medication [16,32,33] as the community becomes acquainted with the disease condition. Because of higher malaria prevalence, caretakers would consider every fever to be malaria and so act without taking a child to the health care facility. Also, because of higher malaria prevalence, there is a possibility that antimalarials are often out of stock and so caretakers decide not to take their children to health care facilities believing they will not get antimalarials. This attitude has been identified in different areas [12,17]. Poor healthcare seeking behavior in areas with malaria prevalence above the national level suggests difficulties in malaria eradication if these fevers are malaria associated [1] or lost opportunity in management of other febrile illnesses in case these fevers are not malaria associated.

The strength of this study lies on representativeness of the data on the population of Tanzania. Also mothers were asked about a history of fever and its management within two weeks preceding the survey, which decreases recall bias. The weakness of this study is the fact that these results are coming from only variables which were collected by THMIS. Important variables which could influence appropriate healthcare seeking behavior like perceived accessibility [34] and the perceived quality of care delivered at health facilities[35] by caretakers were not collected. Also, the cross-sectional nature of the study which inherently cannot infer about causal associations.

## Conclusion

To effectively and appropriately manage and control febrile illnesses, the low proportion of febrile children taken to health facilities by their caretakers should be addressed through frequent advocacy of the importance of appropriate healthcare seeking behavior, using mass media particularly in areas with high malaria prevalence. Multifaceted approach needs to be used in malaria control and eradication as multiple factors are associated with appropriate healthcare seeking behavior.
